# Supplementary material for: Integrated photonics enabling ultra-wideband fibre–wireless communication
Source: Nature. 2026 Feb 18;651(8105):348–55. doi: 10.1038/s41586-026-10172-9 (PMC12979187; doi:10.1038/s41586-026-10172-9)
Supplement: Supplementary file 1 — This file contains Supplementary Figs. 1–32, Supplementary Tables 1–4 and Supplementary References [file 41586_2026_10172_MOESM1_ESM.pdf]

nature portfolio

https://doi.org/10.1038/s41586-026-10172-9

Supplementary information

Integrated photonics enabling ultra-wideband fibre–wireless communication

In the format provided by the authors and unedited

Supplementary Information for

Integrated photonics enabling ultra-wideband fiber-wireless communication

Yunhao Zhang[1,2,5,†], Haowen Shu[2,8,†,*],Yijun Guo[2,†], Peiqi Zhou[3,†], Luyu Wang[4,†], Jianyang Cai[2], Liyuan Yao[2], Linshan Yang[2], Linze Li[4], Tianyu Long[4], Zhouze Zhang[4], Changhao Han[2], Kaihang Lu[6], Yu Sun[7], Zhaopeng Xu[1], Jun Qin[7], Yeyu Tong[6], Zhixue He[1], Xi Xiao[3], Lei Wang[1], Baile Chen[4,*], Shaohua Yu[1,*] and Xingjun Wang[1,2,8,*]

[1]Peng Cheng Laboratory, Shenzhen, China

[2]State Key Laboratory of Photonics and Communications, School of Electronics, Peking University, Beijing, China

[3]National Information Optoelectronics Innovation Center, China Information and Communication Technologies Group Corporation, Wuhan, China

[4]School of Information Science and Technology, ShanghaiTech University, Shanghai, China

[5]School of Electronic and Computer Engineering, Peking University Shenzhen Graduate School, Shenzhen, China

[6]Microelectronic Thrust, the Hong Kong University of Science and Technology (Guangzhou), Guangzhou, China

[7]Key Laboratory of Information and Communication Systems, Beijing Information Science and Technology University, Beijing, China

[8]Frontiers Science Center for Nano-optoelectronics, Peking University, Beijing, China

[†]These authors contributed equally to this work.

Corresponding authors: *haowenshu@pku.edu.cn, *chenbl@shanghaitech.edu.cn, *yush@cae.cn, *xjwang@pku.edu.cn.

CONTENTS

I. **Supplementary note I: Design and Characterization of TFLN modulator** 3

II. **Supplementary note II: Temperature-dependent characterization of the UTC-PD** 7

III. **Supplementary note III: IMDD transmission results** 11

IV. **Supplementary note IV: THz wireless transmission results** 15

V. **Supplementary note V: Performance of complex-biGRU algorithm** 25

References 33

I. SUPPLEMENTARY NOTE I: DESIGN AND CHARACTERIZATION OF TFLN MODULATOR

The bandwidth of the TFLN modulator can be theoretically derived as[1]:

$$\text{EO}\, S_{21} = -10 \times \lg[(1-H)^2 \frac{|S_{21}|^2 - 2 \times |S_{21}| \times \cos(\beta^{\mu}_{opt} L) + 1}{(\ln|S_{21}|)^2 + \cos(\beta^{\mu}_{opt} L)^2}] \tag{1}$$

$$\beta^{\mu}_{opt} = \frac{\omega}{c}(n_m - n_o) \tag{2}$$

$$H = \frac{Z_c - Z_{in}}{Z_c + Z_{in}} \tag{3}$$

where S_{21} is the RF loss in linear scale; β^{μ}_{opt} represents the velocity mismatch term as defined in Eq.2; and H is the impedance mismatch term as defined in Eq.3. To improve the EO bandwidth of the modulator, it is essential to minimize microwave losses while simultaneously achieving optimal velocity matching and impedance matching. In previous studies, RF loss and velocity matching of the electrodes have been thoroughly investigated, whereas impedance matching has often been overlooked, resulting in a lower and non-flat EO response. Moreover, pad areas between modulation electrodes and probes also impact the impedance matching, requiring careful design consideration. In this work, we simultaneously mitigate the RF losses while achieving almost perfect impedance match and velocity match through systematic optimization of electrode and pad parameters.Fig.S1a plots the simulated microwave effective index (n_{m} = 2.188 @ 200 GHz) of the electrodes, which is identical to the optical group index (n_{o} = 2.188 @ 1550 nm). The simulated characteristic impedance of the electrodes (Z_{c} = 50.012 Ω @ 200 GHz) and pads (Z_{pad} = 50.045 Ω @ 200 GHz) are also close to the input impedance (usually Z_{in} = 50 Ω) with a slight deviation <0.1% (Fig.S1b and Fig.S1c).

After device fabrication, we analyze the EO response and impedance matching performance of the modulators. An overview of the experimental setup across the whole frequency bands is also provided. For 0-110 GHz, a coaxial cable with 1.0 mm connector is employed to load the signal. The drive power for S21 characterization can be calculated by subtracting the probe and cable losses from the output power of the LCA, while the drive voltage is derived from the drive power with 50 Ω impedance.(Figure.S2a). For 110-170 GHz and

140-220 GHz, WR-6 and WR-5 rectangular waveguide are employed to deliver the corresponding frequency signal to the modulator. The drive voltage can also be derived from the drive power with 50 Ω impedance, as shown in Figure.S2b and Figure.S2c. The measured EO response from 0-110 GHz is depicted in Figure.S3. The EO S21 exhibits a flat response up to 110 GHz, while the electrical reflection S11 remains mostly below -10 dB, which indicates its impedance matching performance. For frequencies over 110 GHz, we can observe a gradual decline of the EO S21 without significant periodic resonances, indicating that the modulator's electrodes also maintain excellent impedance match and velocity match across 110-220 GHz. We also measure the half-wave voltage of the modulator using 100 kHz triangular sweep as shown in Figure.S4. We obtain a $V_{\pi,\mathrm{LF}}$ of 5.1 V. Based on the measured EO response and $V_{\pi,\mathrm{LF}}$, $V_{\pi,\mathrm{RF}}$ from 1-220 GHz can be calculated according to Eq.2 and $V_\pi L$ at each frequency can be obtained by simply multiplying the modulator length of 5 mm (Figure.S5).

Besides the extraordinary performance, our design also complies with commercial wafer-scale fabrication and are capable for volume production. We have fabricated 4-inch wafer-scale TFLN modulators based on DUV lithography process using the same design parameters. Compared with the EBL modulator, the DUV modulator shows no significant difference in EO response from 110 GHz to 220 GHz, confirming the massive scalability of our solution (Figure.S6).

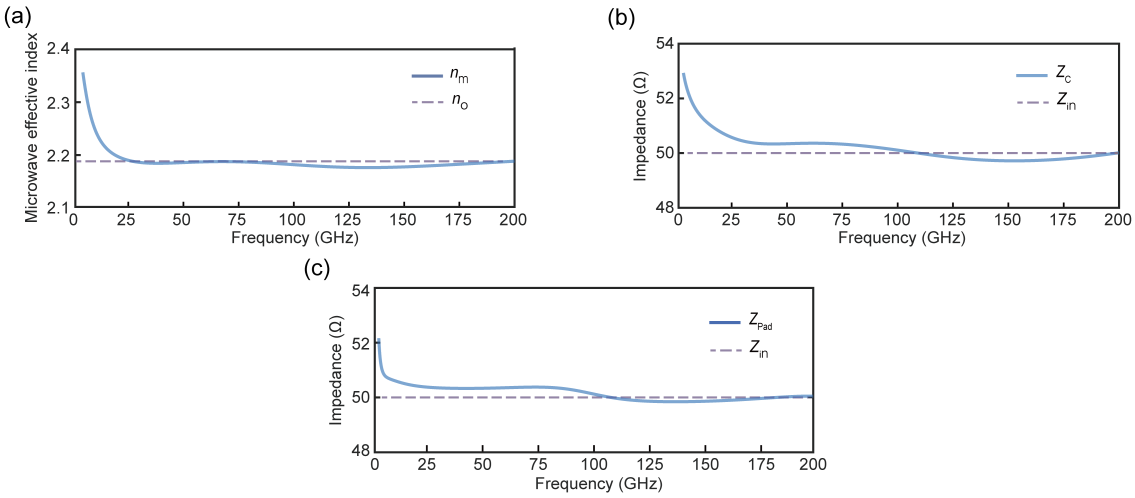

Supplementary Fig. 1: Simulated results for the optimized designs, including (**a**) microwave index of the electrodes, characteristic impedance of the (**b**) electrodes and (**c**) pads.

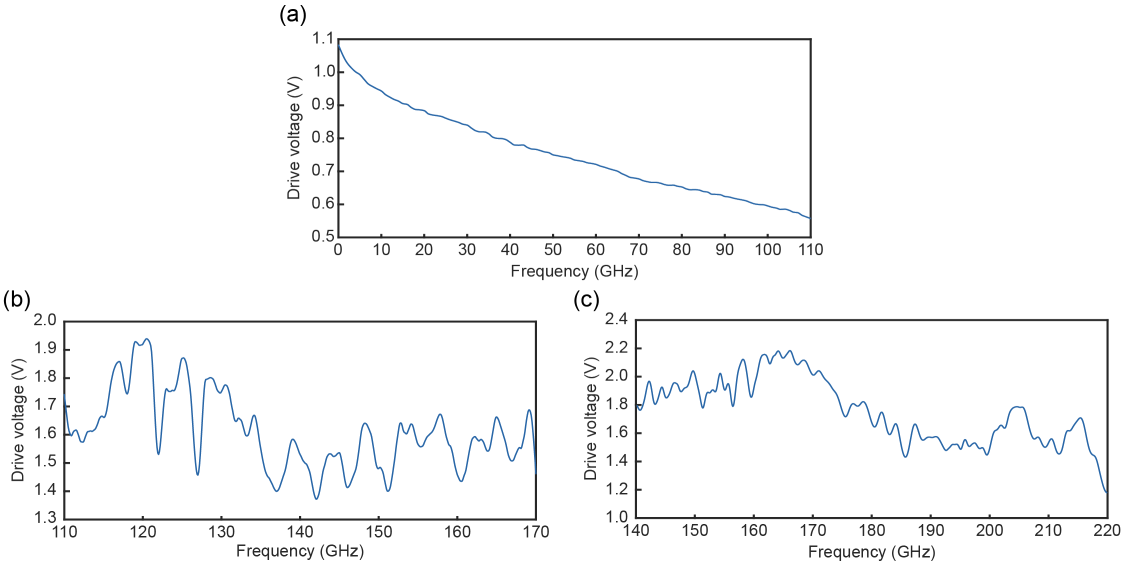

Supplementary Fig. 2: Drive voltage for S21 characterization at the bands of (**a**) 0-110 GHz, (**b**) 110-170 GHz, and (**c**) 140-220 GHz.

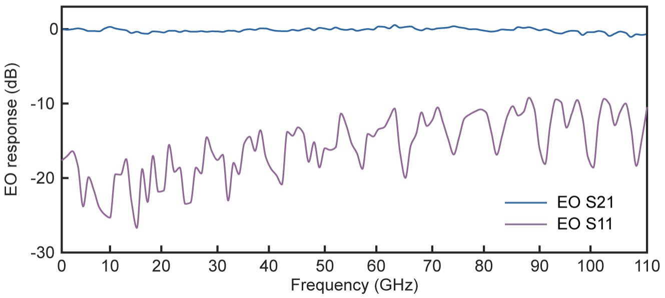

Supplementary Fig. 3: Measured EO response and electrical reflection from 1 GHz to 110 GHz.

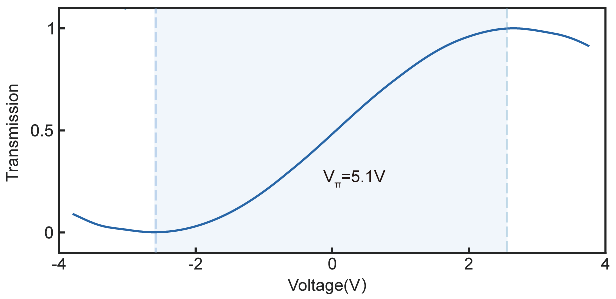

Supplementary Fig. 4: Measured low frequency half-wave voltage.

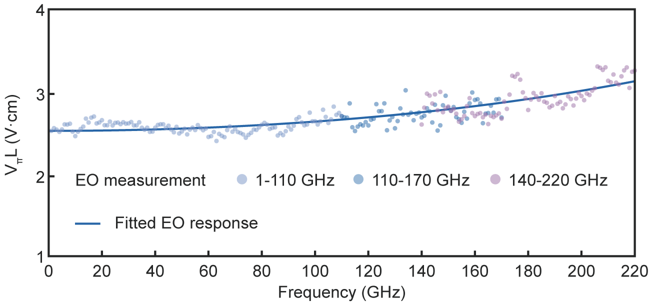

Supplementary Fig. 5: $V_\pi L$ of the ultra-wideband TFLN modulator.

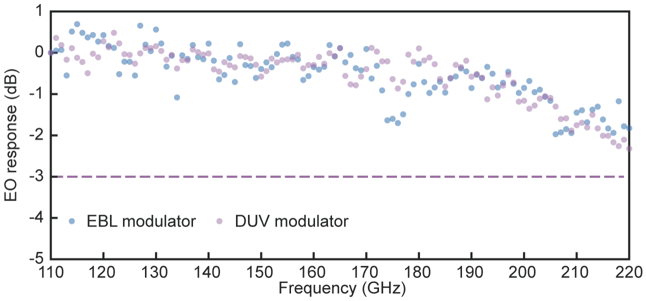

Supplementary Fig. 6: Comparison of 110 GHz - 220 GHz EO response between DUV and EBL process modulators.

II. SUPPLEMENTARY NOTE II: TEMPERATURE-DEPENDENT CHARACTERIZATION OF THE UTC-PD

Temperature-dependent performance is critical for practical applications of PDs, as it directly affects PDs' reliability and robustness under varying environmental conditions. We conduct a series of experiments to characterize the PD's temperature-dependent performance (25–100 °C), covering responsivity, 3 dB bandwidth, output saturation, dark current, and linearity (OIP3). As shown in Figure.S7, the 3 dB bandwidth gradually decreases from 254 GHz at 25 °C to 196 GHz at 100 °C, while maintaining a smooth frequency-response profile over the entire band. The corresponding saturation RF output power measured at 140 GHz, 180 GHz, and 220 GHz (Figure.S8) follows a consistent trend with the frequency-response degradation. From 25 °C to 100 °C, the saturation RF output power drops from 1.26 dBm to -0.44 dBm for 140 GHz, from -1.40 dBm to -3.42 dBm for 180 GHz and from -3.12 dBm to -6.84 dBm for 220 GHz. The dark current increases from 0.02 nA to 1.61 nA at -1.2 V, remaining comparable to that of typical InP PDs at room temperature (Figure.S9). The measured OIP3 at 20 GHz and 40 GHz shows negligible degradation with temperature, indicating that linearity is unaffected (Figure.S10). A detailed summary of measured parameters is provided in Table.SI. These results confirm that the operational window of our modified UTC-PD can be extended up to 100 °C with minor temperature-induced variations, demonstrate robust high-speed and linear operation under cruel environment, and make a critical attribute for uncooled or high-temperature photonic-THz applications.

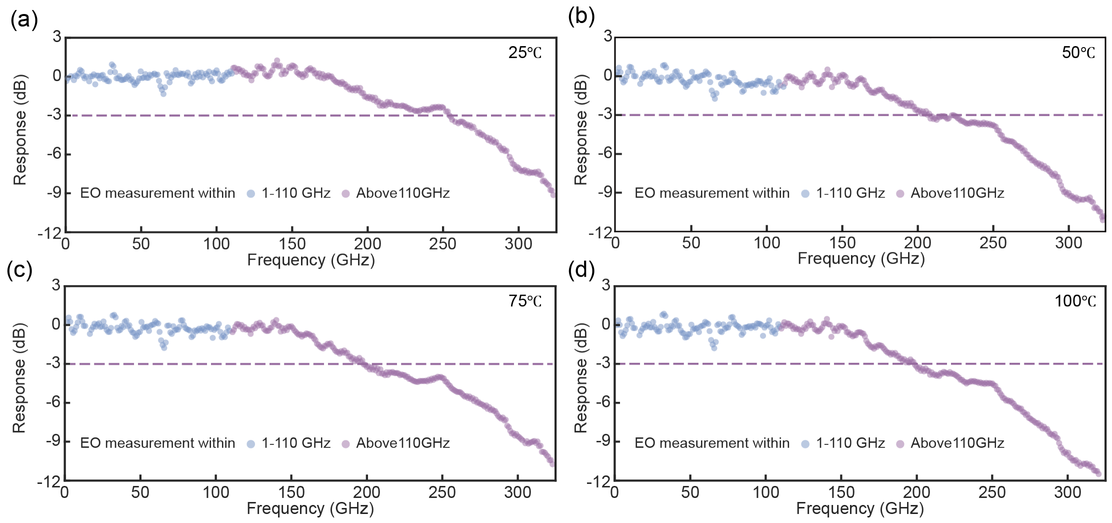

Supplementary Fig. 7: Frequency response of the UTC-PD measured at (**a**) 25 °C, (**b**) 50 °C, (**c**) 75 °C and (**d**) 100 °C.

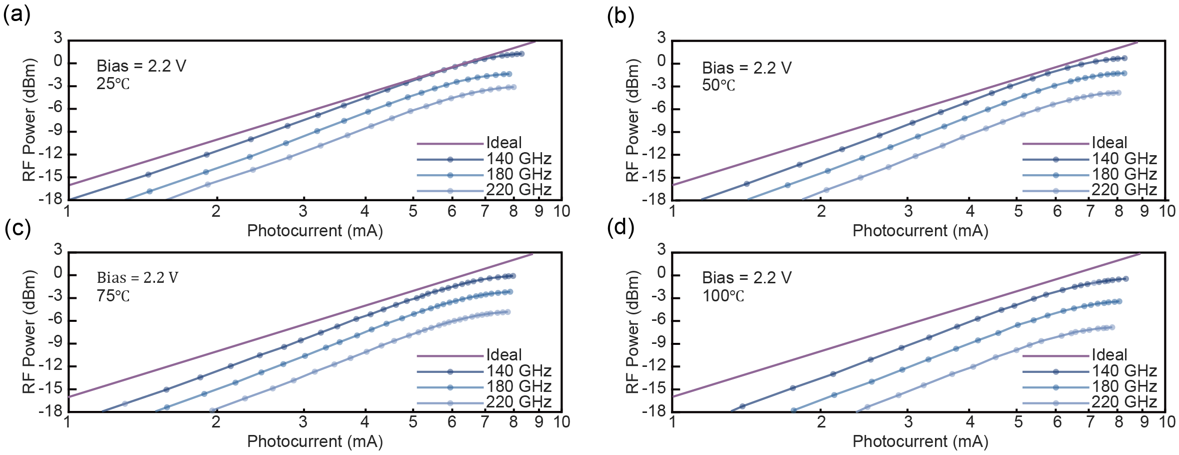

Supplementary Fig. 8: Measured RF output power versus photocurrent at (**a**) 25 °C, (**b**) 50 °C, (**c**) 75 °C and (**d**) 100 °C under a bias of 2.2 V. The solid purple line represents the ideal linear relation.

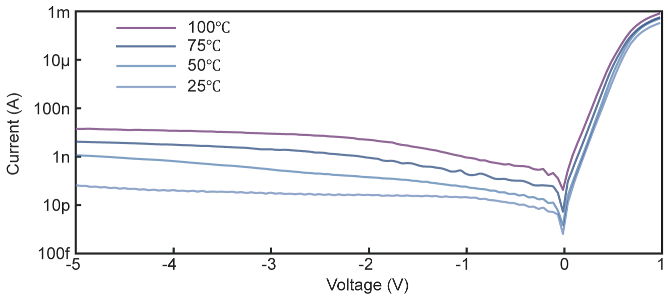

Supplementary Fig. 9: Measured dark current–voltage curves under different temperatures.

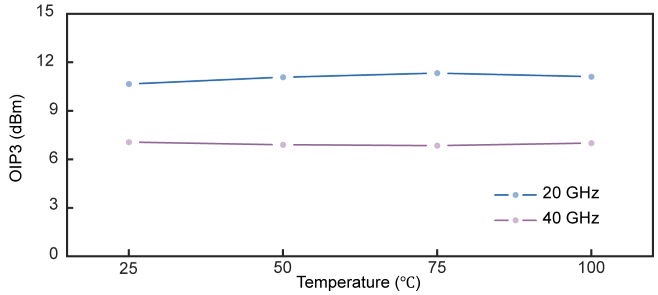

Supplementary Fig. 10: OIP3 of the UTC-PD measured at 20 GHz and 40 GHz under different temperatures.

Supplementary Tab. I: Temperature-dependent performance (25–100 °C) of the modified UTC-PD.

**Temperature (°C)**	**25**	**50**	**75**	**100**
Responsivity (A/W)	0.241	0.218	0.206	0.199
Saturated RF Output Power @ 140 GHz (dBm)	1.26	0.65	-0.09	-0.44
Saturated RF Output Power @ 180 GHz (dBm)	-1.4	-1.32	-2.17	-3.42
Saturated RF Output Power @ 220 GHz (dBm)	-3.12	-3.88	-4.83	-6.84
Dark current @ -1.2 V (nA)	0.02	0.07	0.3	1.61
OIP3 @ 20 GHz (dBm)	10.52	11.08	11.33	11.12
OIP3 @ 40 GHz (dBm)	7.95	6.9	6.85	6.77

III. SUPPLEMENTARY NOTE III: IMDD TRANSMISSION RESULTS

The UWB TFLN modulator exhibits the extraordinary capability for IMDD transmission as we described in the main text. Fig.S11 shows all the eye diagrams directly captured from DSO with no bandwidth compensation DSP. The eye diagrams start to exhibit pattern degradation since 182 Gbaud due to the bandwidth limits of our test equipment but remain visible until 210 Gbaud. We also evaluate the complex-biGRU algorithm's equalization ability across different modulation formats (NRZ, PAM-4) and symbol rates. The transmission results including the eye diagrams and BERs from 168 Gbaud to 256 Gbaud are demonstrated in Fig.S12. The eye diagrams are quite clear and all BERs for all symbol rate are below HD-FEC threshold. Based on these results, we predict BER for the data rates above 500 Gbps by fitting the measured BER results. Fig.S13 shows that when the data rate increases to 680 Gbps, the BER just exceeds over SD-FEC threshold. This result illustrates the remarkable capability and potential of the proposed complex-biGRU powered UWB integrated photonics system in ultra-high speed short-reach communication. With higher-order modulation formats (PAM-8, PAM-16) and larger bandwidth test platforms, single-lane transmission over 1 Tbps is possible based on our approach.

We also analyze the energy efficiency of the fiber communication system. The power consumption breakdown of the system setup is presented in Figure.S14. The power consumption of the fixed wavelength laser is 1 W. An electrical power amplifier is used to amplify the signal generated from AWG consuming 2 W. The TFLN modulator consumes 0.045 W. The EDFA used to boost the modulated optical signal consumes 24 W. The power consumption of PD is 0.0012 W. When applying complex-biGRU algorithm, the DSP consumption is about 42.572 W. The overall system consumption is 69.5732 W, and the energy per bit is 69.6182 W/512 Gbps = 135.97 pJ/bit. Currently, power consumption is primarily concentrated in EDFA and DSP, which can be mitigated through various approaches. High-speed data transmission without optical amplification by utilizing high-power DFB lasers has been demonstrated[2], revealing the potential for future EDFA/SOA-free transmission. Power consumption of the complex-biGRU algorithm can also be further reduced through precision algorithm optimization and using ASIC chips[3].

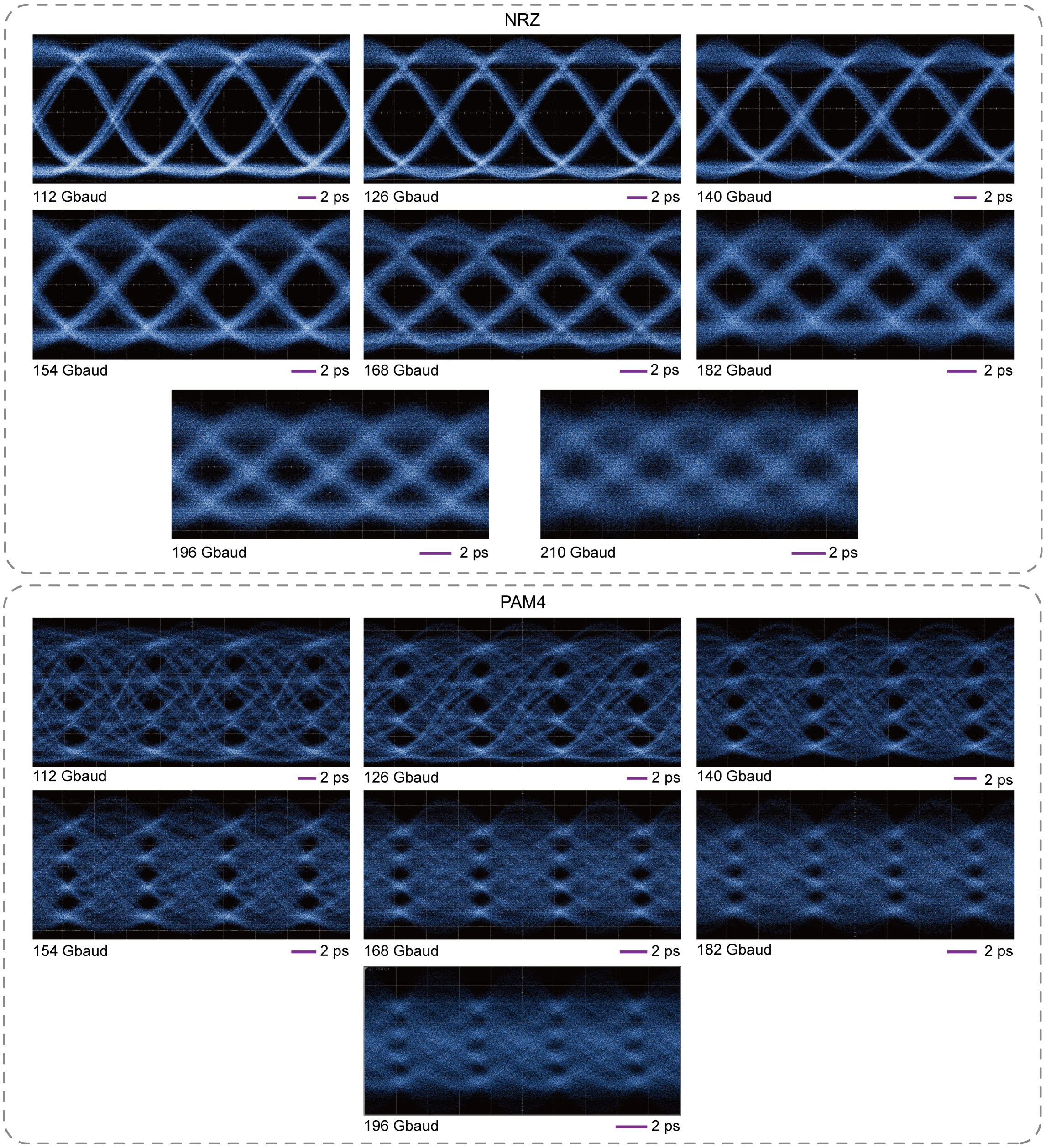

Supplementary Fig. 11: DSP-free IMDD transmission results of NRZ and PAM-4 signals at different symbol rate.

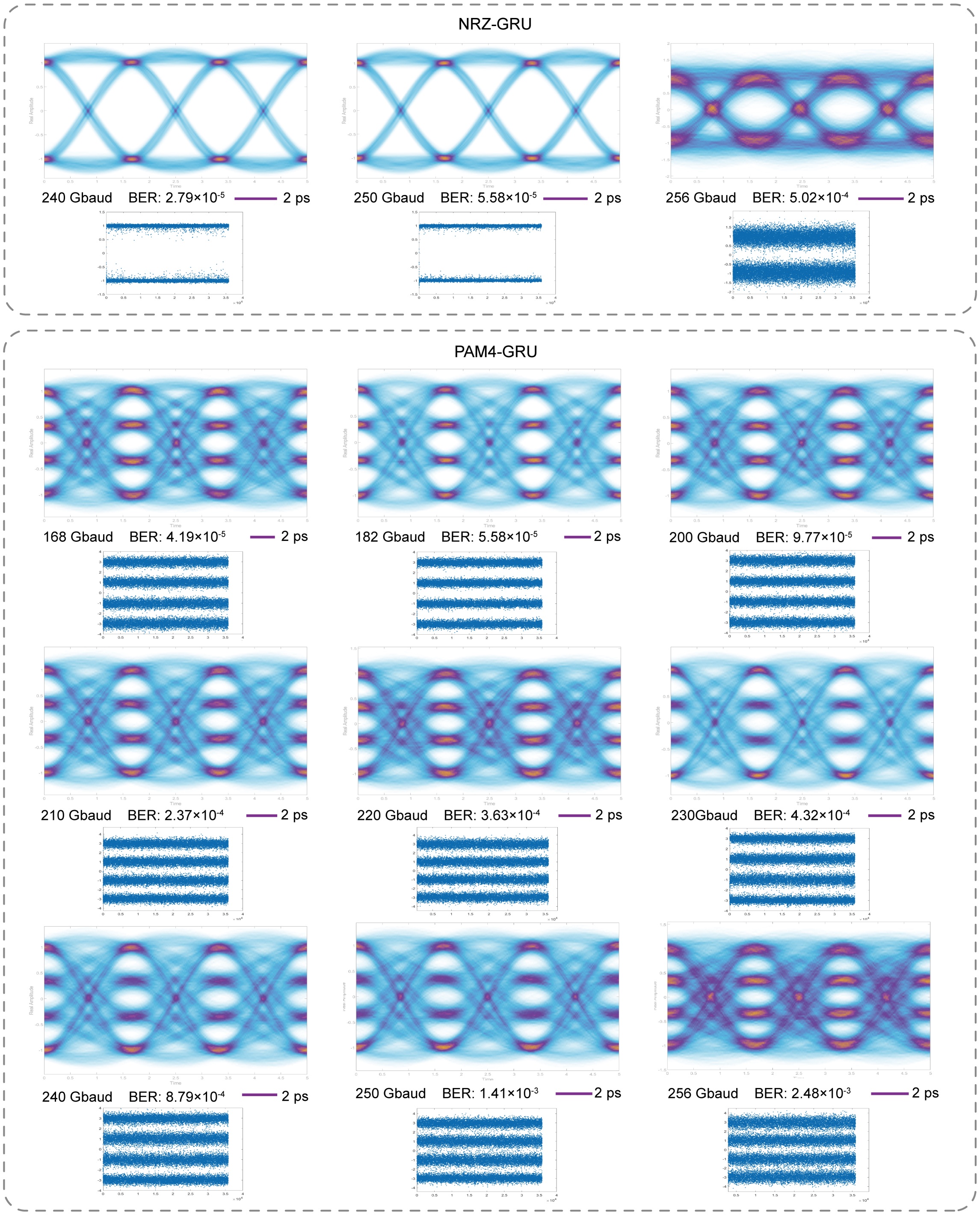

Supplementary Fig. 12: Complex-biGRU based IMDD transmission results of NRZ and PAM-4 signals at different symbol rate.

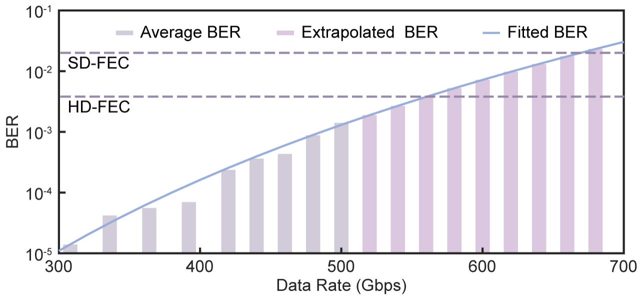

Supplementary Fig. 13: The fitting BER curve for complex-biGRU based PAM-4 transmission.

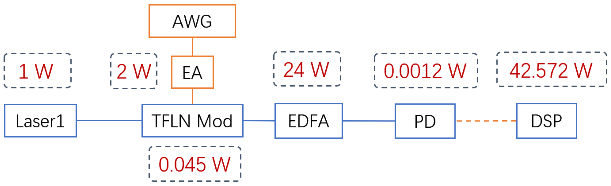

Supplementary Fig. 14: Schematic diagram of the fiber communication system power consumption.

IV. SUPPLEMENTARY NOTE IV: THZ WIRELESS TRANSMISSION RESULTS

Besides short-reach interconnection, the proposed UWB integrated photonics system also significantly enhances the wireless communication performance as we described in the main text. Fig.S15 displays all the constellation diagrams obtained in the experiment with baseline DSP. The system can support up to 90 Gbaud and 240 Gbps THz wireless transmission within SD-FEC threshold. With the help of the complex-biGRU algorithm, the transmission rate can be further increased beyond 100 Gbaud and 400 Gbps (Fig.S16). The complex-biGRU algorithm brings about 67% enhancement in data rates. We also perform non-linear fitting based on the 16-QAM transmission results to predict BER for higher speed. Fig.S17 shows that the BER slightly exceeds over SD-FEC threshold when the data rate increases to 500 Gbps. Currently, the system's performance is mainly limited by the analog bandwidth of the test equipment. We firmly believe that our UWB system would support a higher single-channel data rate and symbol rate by employing better equipment in our future demonstration.

In addition to the transmission rate, we also characterize several other critical metrics in the wireless system. For the sensitivity of our THz receiver, free space path loss (FSPL) can be calculated using the following equation:

$$FSPL = 20 \times \lg(d) + 20 \times \lg(f) + 32.44 - G_t - Gr \tag{4}$$

where d is the propagation distance of signal in meters, f is the signal frequency in GHz, and G_t and G_r are the gains of transmitter and receiver antenna. By subtracting the FSPL from the output power of the PD, the received power at the input of the Rx amplifier can be calculated. The emitted power of the UTC-PD can be precisely regulated by adjusting the incident optical power. The BER curve of 70 Gbaud QPSK transmission under different received power is obtained (Figure.S18) for example. It can be seen that BER decreases with increasing received power. When the received power exceeds -23 dBm, the BER performance also deteriorates due to amplifier saturation.

For the frequency stability of our system, we analyze the Tx LO frequency-set accuracy, Rx zero-IF alignment error and capture range. Due to the influence of spontaneous emission and temperature drift, the frequency of the laser is not absolutely stable and exhibits frequency jitter. To quantify the Tx LO frequency-set accuracy, we characterize the

free-running ECLs used in our experiment at Tx sides. As illustrated in Figure.S19a, the beat signal generated by two ECLs is captured by a commercial PD and analyzed using an electrical spectrum analyzer (ESA). We measure the frequency stability of the beat signal within 30 minutes and the beat signal drifts about 21 MHz as shown in Figure.S19b.

For Rx zero-IF alignment error, we employ the experimental setup in Figure.S20a for characterization. The beat signal generated by two Tx ECLs is loaded to an off-the-shelf modulator. The Rx LO beats with the modulated sideband via a second PD, and the zero-IF alignment error is analyzed through the ESA. We test the frequency drift within 30 minutes and the maximum drift range is about 43 MHz, as shown in Figure.S20b.

Although there is slight frequency drift between the free-running lasers, it does not affect the performance of the high-speed wireless communication system due to the wide capture range. We detune the Rx LO from zero-IF and demodulate the 70 Gbaud QPSK signal. The BER deteriorates with the frequency offset increasing as illustrated in Figure.S21. It can be seen that the system maintains acceptable performance with a capture range up to 8 GHz, far exceeding the 43 MHz frequency drift of the lasers. From the results one can infer, for the ultrabroadband signal transmission, tens of MHz frequency drift of the lasers have negligible impact.

To mitigate the impact of frequency drift on the system's performance, we can employ narrow-linewidth lasers with temperature control modules to enhance the stability of the beat signal. Various approaches have also been proposed to track and recover the frequency jitter in the transmission link, including self-coherent[4], time-domain pilots[5] and residual carrier modulation[6]. While these methods are demonstrated in fiber communication, they can be seamlessly transferred to wireless communication, simultaneously compensating frequency jitter of beat signals at both Tx and Rx ends.

For energy efficiency analysis of the system, we present the power consumption breakdown of the high-speed wireless communication setup as illustrated in Figure.S22. At the transmitter side, the fixed wavelength laser served as the signal carrier consumes 1 W. The power consumption of IQ modulator is 7.92 W, including drivers and automatic bias control. The tunable ECL serves as the Tx LO with a power consumption of 50 W. The EDFA amplifies the mixed signals with a power consumption of 24 W. The amplified optical signal is then sent to the modified UTC-PD, consuming only 0.0044 W (2.2 V, 2 mA). At the receiver side, the signal is amplified by a low-noise amplifier (0.054 W) before sent into the

TFLN modulator (0.045 W) for THz-to-optical conversion. The high-power laser driving the TFLN modulator is 20 W. The EDFA power consumption at the receiver side is 24 W. Another tunable ECL acting as the Rx LO consuming 50 W. The power consumption of the complex-biGRU DSP algorithm for demodulating signals is 50.7 W. The overall link consumption is 227.7234 W. And the energy per bit is 227.7234 W/400 Gbps = 569.3 pJ/bit.

It is worth noting that in the current system, power consumption is primarily concentrated in tunable ECLs, EDFA and DSP. These high-power devices can be replaced in the future (Figure.S23): DFB lasers can replace ECLs, with power consumption as low as 0.5 W. SOA can replace EDFA, with power consumption of only 0.8 W[6–8]. Thus, overall system power consumption is projected to decrease to 62.3234 W, and the energy per bit will drop to 155.8 pJ/bit. Furthermore, power consumption of the complex-biGRU algorithm can also be reduced through precision algorithm optimization and using ASIC chips[3].

Besides characterizing the high-speed wireless transmission system, we also evaluate the performance of the multi-channel video transmission system. We use an ESA to get the spectrum of the video signal as shown in Figure.S24. The signal demonstrates a real-time bandwidth of 100 MHz with over 20 dB SNR, which also indicates an adjacent-channel isolation over 20 dB. With a channel spacing of 1 GHz, the guard interval can be calculated as 1000 MHz - 100 MHz = 900 MHz.

The BER performance of each channel is also assessed. According to the specification of HDMI 2.1[9], character (with 18 bits in each character) error rates of 10^{-4} or higher are readily detectable by visual inspection. Our demonstration achieves consecutive and clear live video transmission across all 86 channels with no visible error, indicating that the BER of each channel remains below 10^{-4} / 18 = 5.5×10^{-6}.

We also present the power consumption breakdown of the video transmission system as shown in Figure.S25. At the transmitter side, the switch served for signal routing consumes 43 W. The power consumption of two optical modules is 1.8 W each. The tunable ECL serves as the Tx LO with a power consumption of 50 W. The EDFA amplifies the mixed signals with a power consumption of 24 W. The amplified optical signal is then sent to the modified UTC-PD, consuming only 0.0044 W (2.2 V, 2 mA). At the receiver side, the signal is amplified by a low-noise amplifier (0.054 W) before sent into the TFLN modulator (0.045 W) for THz-to-optical conversion. The high-power laser driving the TFLN modulator is 20 W. The EDFA power consumption at the receiver side is 24 W. Two optical modules at

the receiver side consumes 1.8 W each. And the power consumption of the Rx switch is 43 W. The overall system consumption is 211.3034 W. It is worth noting that in the current system, two of the major power consumption equipment are tunable ECL and EDFAs. These high-power devices can be replaced in the future: DFB lasers can replace ECLs, with power consumption as low as 0.5 W. SOA can replace EDFA, with power consumption of only 0.8 W[6–8].

To meet the communication distance requirements of the envisioned applications, we also verify the system's performance with the transmission distance increasing to 4 m. The experimental setup shown in Figure.S26. We replace the origin horn antennas with high-gain lens antennas. Figure.S27 depicts the transmission results of the 96 Gbaud QPSK and 76 Gbaud 16-QAM based on baseline DSP. After signal recovering, BER results meet the 20% SD-FEC threshold requirement. With the help of complex-biGRU algorithm, the transmission rate can be further increased to 100 Gbaud and 400 Gbps (Figure.S28). Distinguishable constellation diagrams of the 100 Gbaud QPSK and 100 Gbaud 16-QAM are observed and BER results maintains below the 20% SD-FEC threshold. In addition to high-speed THz communication, 8K real-time video transmission at a 4-meter distance has also been demonstrated, as shown in the Supplementary Video.

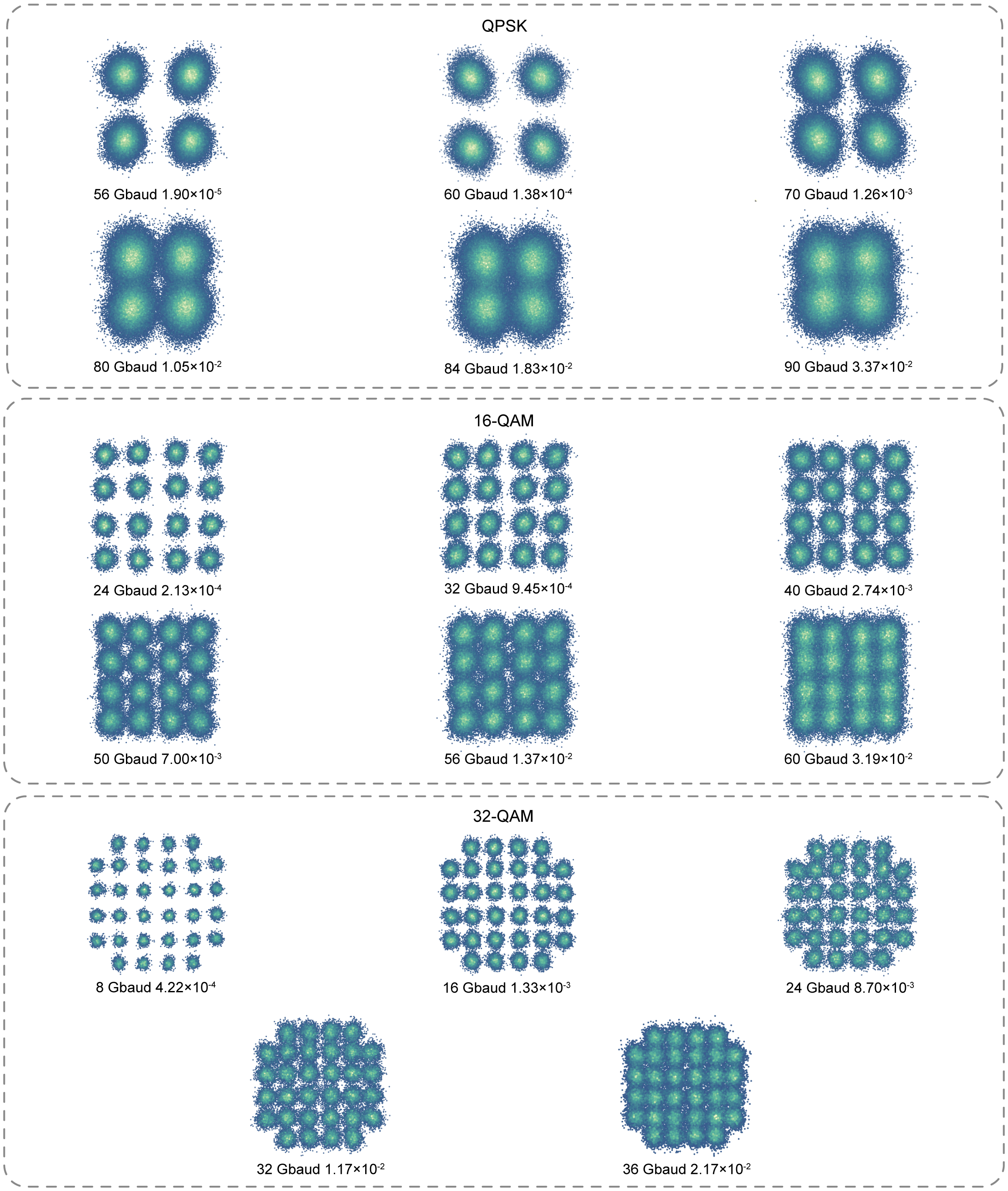

Supplementary Fig. 15: Baseline DSP based wireless transmission results of QPSK, 16-QAM and 32-QAM signals at different symbol rate.

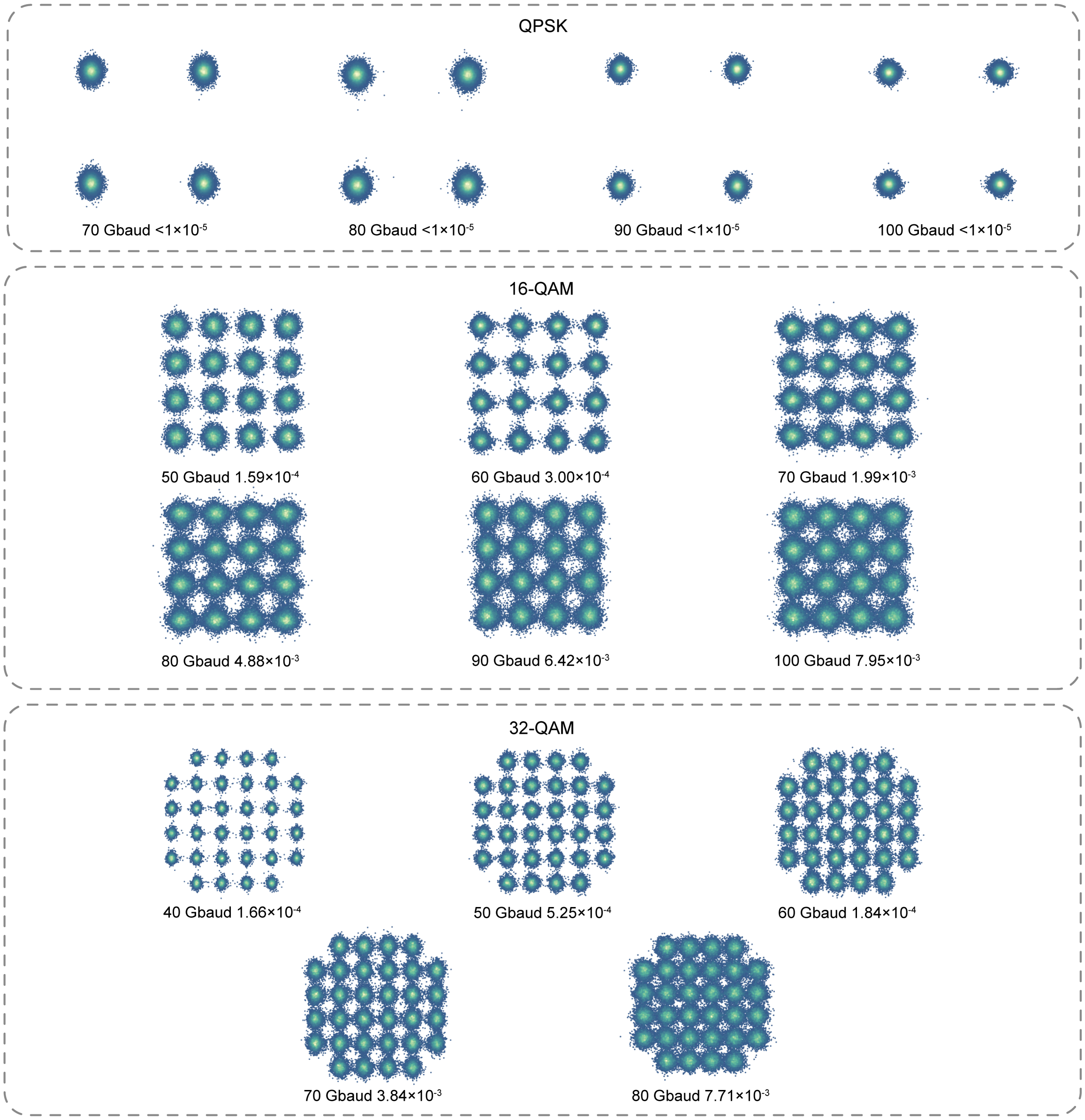

Supplementary Fig. 16: Complex-biGRU based wireless transmission results of QPSK, 16-QAM and 32-QAM signals at different symbol rate.

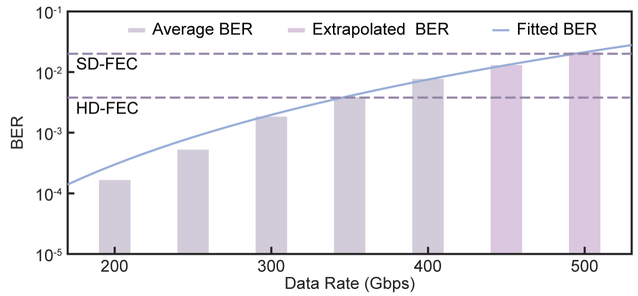

Supplementary Fig. 17: The fitting BER curve for complex-biGRU based 16-QAM transmission.

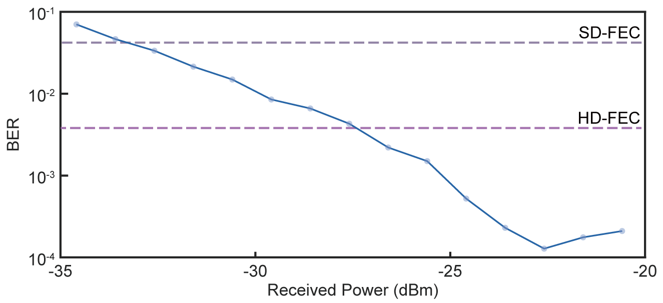

Supplementary Fig. 18: BER curves of different receiving power.

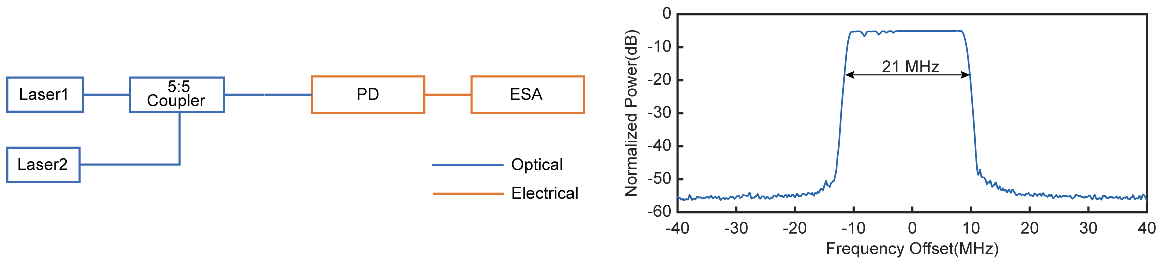

Supplementary Fig. 19: (**a**) Schematic diagram and (**b**) 30 minutes results of the Tx LO frequency-set accuracy experiment.

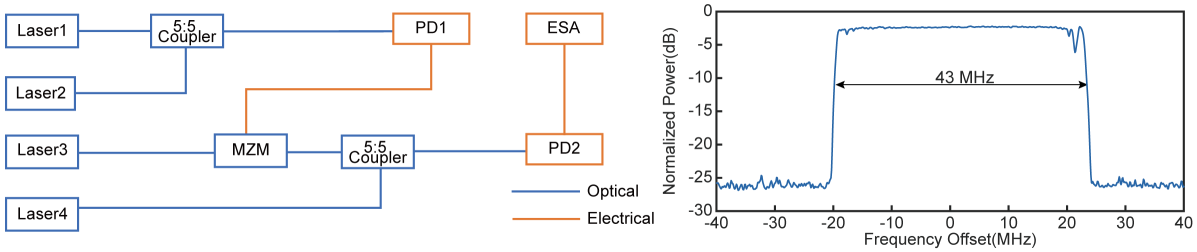

Supplementary Fig. 20: (**a**) Schematic diagram and (**b**) 30 minutes results of the Rx zero-IF alignment error experiment.

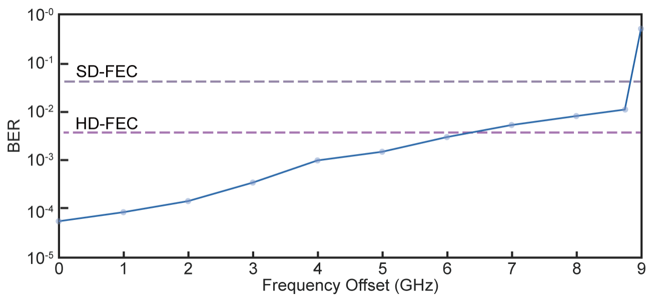

Supplementary Fig. 21: Capture range of the proposed high-speed wireless communication system.

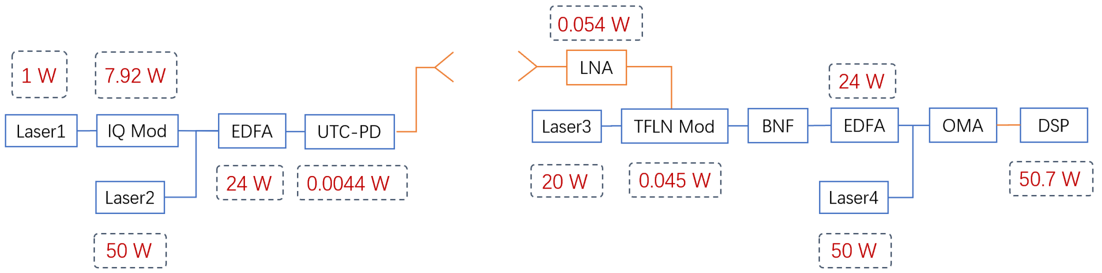

Supplementary Fig. 22: Schematic diagram of the high-speed wireless communication system power consumption.

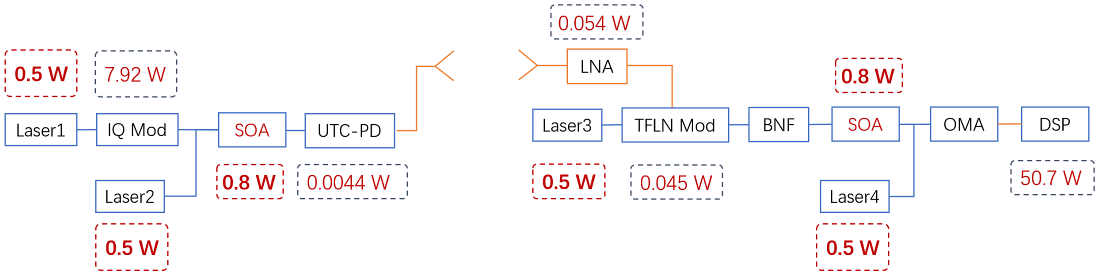

Supplementary Fig. 23: Schematic diagram of the future high-speed wireless communication system power consumption.

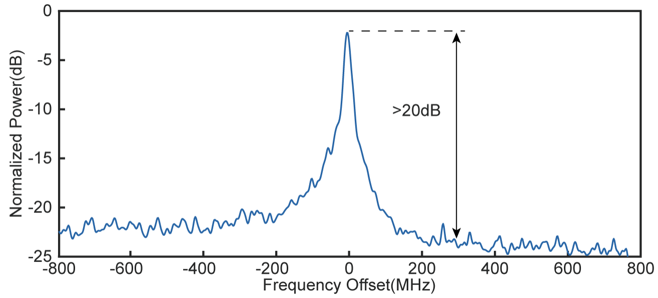

Supplementary Fig. 24: Spectrum of the video signal.

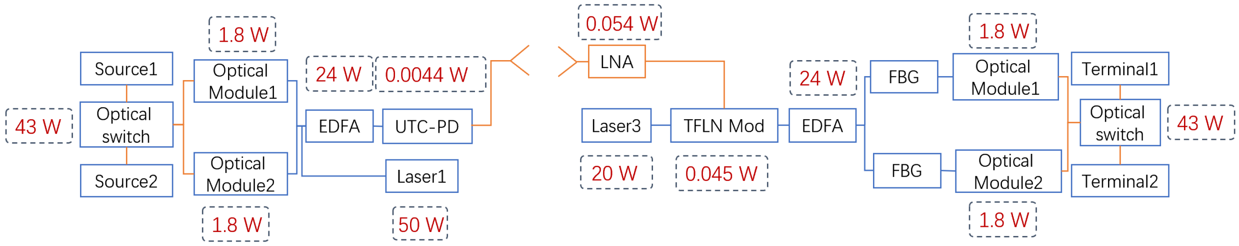

Supplementary Fig. 25: Schematic diagram of the video transmission system power consumption.

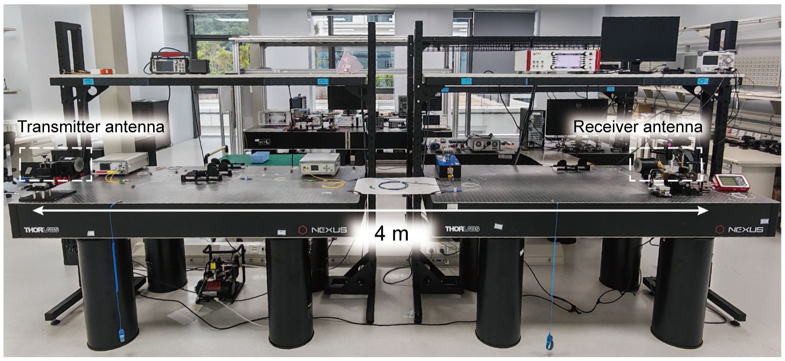

Supplementary Fig. 26: Photograph of the 4-m experimental set-up.

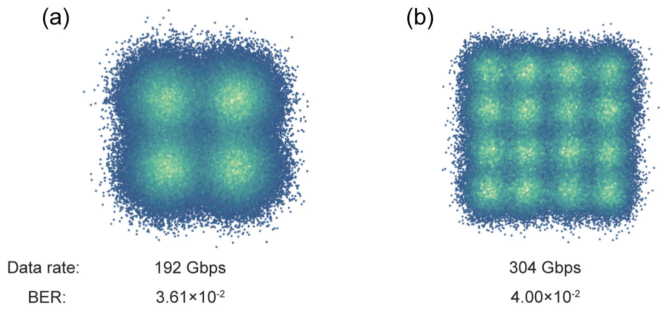

Supplementary Fig. 27: Constellation diagrams and BER results for (**a**) 96 Gbaud QPSK and (**b**) 76 Gbaud 16-QAM with baseline DSP method.

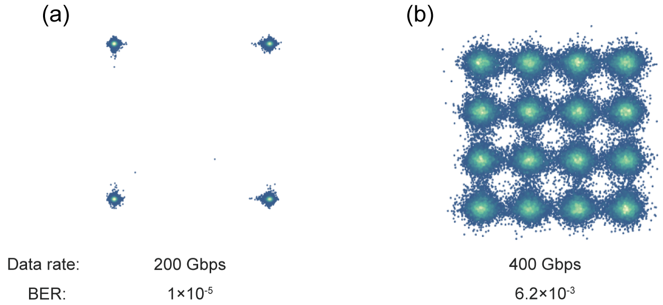

Supplementary Fig. 28: Constellation diagrams and BER results for (**a**) 100 Gbaud QPSK and (**b**) 100 Gbaud 16-QAM with complex-biGRU algorithm.

V. SUPPLEMENTARY NOTE V: PERFORMANCE OF COMPLEX-BIGRU ALGORITHM

The proposed complex-biGRU algorithm not only reduces the BER but also alleviates the signal distribution distortion commonly observed in other NN-based equalization approaches. Fig.S29 shows the constellation diagrams of 25 Gbaud 16-QAM with different equalization methods. It can be observed that either the use of DNN network or conventional ReLU activation function tends to squeeze the signals towards the boundaries of constellation diagrams, resulting in severe signal distortion. Such phenomenon is called 'jail window' effect[10]. Our approach effectively eliminates this undesirable effect, demonstrating constellation points that conform to a 2D Gaussian distribution.

To comprehensively demonstrate the performance of our proposed complex-biGRU algorithm, we compare it with other commonly used algorithms in terms of computational complexity, power efficiency, latency, and equalization capability.

For complexity analysis, we compare the complex-biGRU algorithm with two typical nonlinear equalization methods — Volterra nonlinear equalizers (VNLE) and neural networks (NN). Considering that both VNLEs and NNs are inherently formulated in terms of matrix or tensor operations, where the dominant computational burden arises from multiplications between the weight matrices and the input data, we adopt the multiply–accumulate operation (MAC) as a representative metric for complexity comparison. A single MAC corresponds to one multiplication followed by one accumulation within the matrix multiplication process. Table.SII summarizes the computational complexity of several algorithms. For VNLE, L_n refers to the n-th order memory length. For two-layer deep neural networks (DNN), n_0 refers to the size of input layer, n_3 represents the size of output layer, and n_1/n_2 denotes the number of neurons in the first and second hidden layers respectively. For GRU based algorithms, n_E refers to the input size of the GRU layer, and n_H represents the number of GRU units that used in the hidden layer. To provide a more intuitive comparison of complexities, we calculated the floating-point operations (FLOPs) per symbol for the three algorithms involved in the comparative analysis. For the VNLE algorithm, we employ a second-order Volterra model (i.e., N=2), and the order memory length is set to 80. The

number of MACs can be calculated by:

$$C_{VNLE} = \sum_{n=1}^{N} \frac{(L_n - 1 + n)!}{(n-1)!(L_n - 1)!} = \sum_{n=1}^{N} \frac{(80 - 1 + n)!}{(n-1)!(80-1)!} = 6560$$

So, the required FLOPs per symbol of VNLE are $6560 \times 2 = 13120$. For the DNN algorithm, the size of input layer, first/second hidden layer, and output layers are 30, 40, 40 and 1 respectively. The number of MACs can be then calculated as:

$$C_{DNN} = n_0 n_1 + n_1 n_2 + n_2 n_3 = 30 \times 40 + 40 \times 40 + 40 \times 1 = 2840$$

So, the required FLOPs per symbol of DNN are $2840 \times 2 = 5680$. For the complex-biGRU algorithm, the number of GRU units is set to 200. In wired transmission scenario, only one branch of the network is activated and the input size of the GRU layer is $2^{15} - 1$. While in the wireless transmission scenario, the input size of the GRU layer is 10^5. The number of MACs can be then calculated as:

$$C_{complex-biGRU,wired} = 2 \times 3 n_H (n_E + n_H) = 2 \times 3 \times 200 \times (200 + 2^{15} - 1) = 3.956 \times 10^7$$

$$C_{complex-biGRU,wireless} = 2 \times 2 \times 3 n_H (n_E + n_H) = 2 \times 2 \times 3 \times 200 \times (200 + 10^5) = 2.405 \times 10^8$$

So, the required FLOPs per symbol of complex-biGRU are $(2 \times 3.956 \times 10^7)$ / $(2^{15} - 1) \approx 2414$ for wired scenario and $(2 \times 2.405 \times 10^8)$ / $10^5 \approx 4809$ for wireless scenario. We can see that the number of FLOPs of the complex-biGRU algorithm is at a comparable order of magnitude to that of other neural network algorithms or the VNLE algorithm, indicating that the complex-biGRU algorithm does not increase computational complexity.

We also evaluate the complex-biGRU algorithm's performance under different network complexities. Specifically, we investigate complex-biGRU equalization with different number of hidden GRU units for 50 Gbaud 16-QAM and 70 Gbaud 16-QAM transmission, as depicted in Figure.S30. As the number of GRU units increase, the algorithm's channel equalization capability gradually improves, with the complete channel response being progressively modeled, leading to a reduction in BER. However, when the number of GRU units reached a certain threshold (i.e., 200 GRU units in this case), the BER ceased to decrease further, exhibiting a saturation region. Continued increase in GRU units will lead to overfitting and higher power consumption. Thus, our strategy is to employ the minimum GRU units of the saturation region at each speed to balance performance and efficiency.

For power consumption analysis, we introduce the equalizer power consumption per bit (EPCpB) as a comparison metric for different speeds, which is defined as:

$$\text{EPCpB} = \text{Power Consumption (W)} \ / \ \text{Data Rate (Gbps)}$$

A smaller EPCpB value indicates a lower power consumption per bit. We evaluate and compare the power consumption of traditional nonlinear algorithms and other AI equalizers as listed in Table.SIII. The maximum transmission rate below SD-FEC/HD-FEC threshold is selected as the reference speed for comparison. It can be seen that in simple channel environments, the complex-biGRU algorithm achieves higher transmission rates with comparable power consumption. For the complex channel circumstances such as wireless communication or ultra-high-speed communication, the complex-biGRU algorithm does not significantly increase the EPCpB and even reduce it due to a much larger data rate. In other words, it is worthwhile to use higher equalizer power consumption in exchange for an improvement in transmission speed based on AI equalizers.

We also calculate and compare the latency of different algorithms. The latency introduced by the equalizer depends on several factors, primarily including inference latency and data transfer latency. Inference latency refers to the time required for an algorithm to process an input signal and generate the corresponding output, which can be calculated by:

$$L_{inference} = \text{Total Operations} \ / \ \text{Hardware Throughput}$$

Data transfer latency refers to the time required to move input data from memory to the processing unit and the output from the processing unit back to memory or to the next processing stage, which can be estimated as:

$$L_{transfer} = \text{Data Size} \ / \ \text{Memory Bandwidth}$$

Therefore, the total delay can be calculated as the sum of the inference latency and data transfer latency.

$$L_{total} = L_{inference} + L_{transfer}$$

Based on the equations above, we calculate and summarize the average latency per symbol of each equalization algorithm under different transmission scenarios using different hardware in Table.SIV. We can see that the total latency of different algorithms is comparable and

using GPU can significantly reduce the total latency. It's worth noting that the latency here only includes the time that equalizer takes to process a single symbol. The overall system latency also includes the time consumed by resampling, clock synchronization, signal decision-making and other signal processing procedures. Currently, the latency is measured and calculated based on a generalized platform, which can be further reduced through precision algorithm optimization and employing ASIC based hardware.

To more intuitively compare the equalization capacities of different algorithms, we employ various DSP algorithms to equalize the same set of data collected under different scenarios and rates. In wired transmission, we evaluate the performance of DFE, VNLE, DNN and complex-biGRU for high-speed NRZ and PAM-4 signals as illustrated in Figure.S31. The results demonstrate that, compared to DFE and VNLE, complex-biGRU can achieve significant performance improvements for NRZ and PAM-4 signals, showing an improvement of one or two orders of magnitude in BER. For wireless transmission scenarios, the comparative performance of VNLE and complex-biGRU for high-speed 16-QAM and 32-QAM signals is evaluated, with the results presented in Figure.S32. The results reveal that complex-biGRU also yields remarkable enhancements in BER over VNLE, especially for high-speed signal with large signal bandwidth.

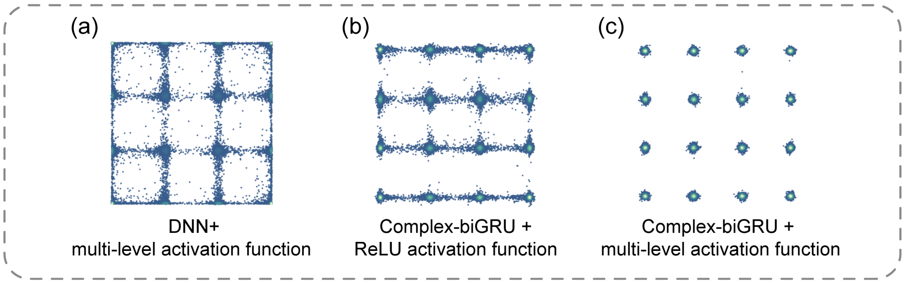

Supplementary Fig. 29: Constellation diagrams of 25 Gbaud 16-QAM based on the equalization using (**a**) DNN with multi-level activation function, (**b**) Complex-biGRU with ReLU activation function and (**c**) Complex-biGRU with multi-level activation function.

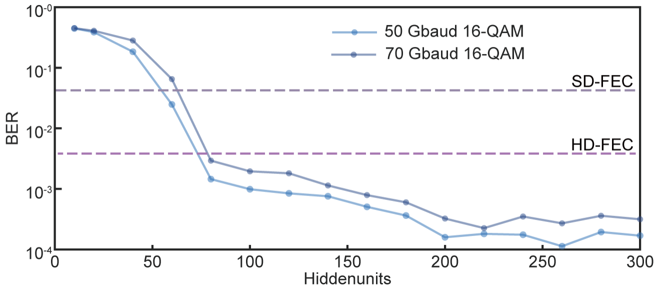

Supplementary Fig. 30: BER curves of different GRU numbers in the hidden layer.

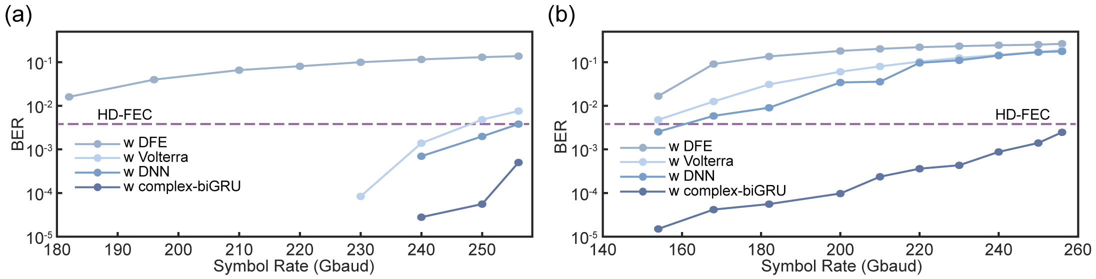

Supplementary Fig. 31: BER performance of DFE, VNLE, DNN and complex-biGRU in wired scenarios for (**a**) NRZ and (**b**) PAM-4 signal transmission.

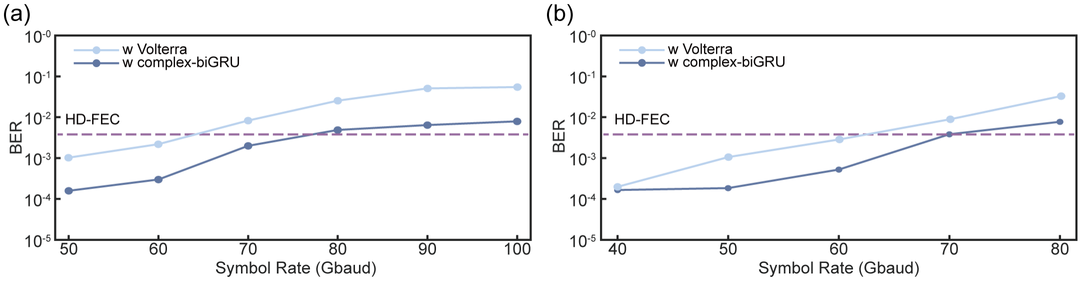

Supplementary Fig. 32: BER performance of VNLE and complex-biGRU in wireless scenarios for (**a**) 16-QAM and (**b**) 32-QAM signal transmission.

Supplementary Tab. II: Comparison of computational complexity among different algorithms.

**Algorithm**	**MAC**
VNLE	$\sum_{n=1}^{N} \frac{(L_n-1+n)!}{(n-1)!(L_n-1)!}$
two-layer DNN	$n_0 n_1 + n_1 n_2 + n_2 n_3$
GRU	$3n_H(n_E + n_H)$
bi-GRU	$2 \times 3n_H(n_E + n_H)$
complex-biGRU	$2 \times 2 \times 3n_H(n_E + n_H)$

Supplementary Tab. III: Comparison of power consumption for different algorithms under different scenarios.

**Equalizer**	**Power (W)**	**Signal format**	**Speed (Gbaud)**	**BER threshold**	**EPCpB (nJ/bit)**
VNLE	∼40.09	NRZ	240	HD-FEC	0.1670
VNLE	∼42.25	PAM-4	168	SD-FEC	0.1257
DNN	∼12.79	NRZ	250	HD-FEC	0.0512
DNN	∼17.15	PAM-4	154	HD-FEC	0.0557
complex-biGRU	∼38.15	NRZ	256	HD-FEC	0.1490
complex-biGRU	∼42.57	PAM-4	256	HD-FEC	0.0831
VNLE	∼71.03	16-QAM	80	SD-FEC	0.2220
VNLE	∼71.23	32-QAM	80	SD-FEC	0.1781
complex-biGRU	∼50.64	16-QAM	70	HD-FEC	0.1808
complex-biGRU	∼50.78	32-QAM	60	HD-FEC	0.1693

Supplementary Tab. IV: Comparison of equalizer latency for different algorithms under different scenarios.

Scenario	Equalizer	Inference latency	Data transfer latency	Total latency
Wired	VNLE	CPU: 15.530 ns	DDR: 0.100 ns	CPU: 15.630 ns
		GPU: 0.901 ns	GDDR: 0.031 ns	GPU: 0.932 ns
	DNN	CPU: 6.724 ns	DDR: 0.100 ns	CPU: 6.824 ns
		GPU: 0.390 ns	GDDR: 0.031 ns	GPU: 0.421 ns
	complex-biGRU	CPU: 2.858 ns	DDR: 0.100 ns	CPU: 2.958 ns
		GPU: 0.166 ns	GDDR: 0.031 ns	GPU: 0.197 ns
Wireless	VNLE	CPU: 15.530 ns	DDR: 0.090 ns	CPU: 15.620 ns
		GPU: 0.901 ns	GDDR: 0.028 ns	GPU: 0.929 ns
	complex-biGRU	CPU: 5.693 ns	DDR: 0.090 ns	CPU: 5.783 ns
		GPU: 0.330 ns	GDDR: 0.028 ns	GPU: 0.358 ns

[1] Zhang, Y. *et al.* Systematic investigation of millimeter-wave optic modulation performance in thin-film lithium niobate. *Photonics Research* **10**, 2380–2387 (2022).

[2] Ostrovskis, A. *et al.* Optical Amplification-Free 400 Gbps Net Bitrate Links with a TFLN-based Transmitter. In *Optical Fiber Communication Conference*, M1G–1 (Optica Publishing Group, 2025).

[3] Aguirre, F. *et al.* Hardware implementation of memristor-based artificial neural networks. *Nature communications* **15**, 1974 (2024).

[4] Shieh, W., Sun, C. & Ji, H. Carrier-assisted differential detection. *Light: Science & Applications* **9**, 18 (2020).

[5] Olsson, S. L. *et al.* Record-high 17.3-bit/s/Hz spectral efficiency transmission over 50 km using probabilistically shaped PDM 4096-QAM. In *2018 Optical Fiber Communications Conference and Exposition (OFC)*, 1–3 (IEEE, 2018).

[6] Fang, X. *et al.* Overcoming laser phase noise for low-cost coherent optical communication. *Nature Communications* **15**, 6339 (2024).

[7] Tao, Z. *et al.* Ultrabroadband on-chip photonics for full-spectrum wireless communications. *Nature* 1–8 (2025).

[8] Li, W. *et al.* 100-km polarization-orthogonal self-homodyne coherent wdm transmission using nonlinearity suppressed soa. In *CLEO: Science and Innovations*, STu4G–5 (Optica Publishing Group, 2023).

[9] High-Definition Multimedia lnterface Specification Version 2.1. *HDMl Forum* (2017).

[10] Freire, P. J. *et al.* Neural Networks-Based Equalizers for Coherent Optical Transmission: Caveats and Pitfalls. *IEEE Journal of Selected Topics in Quantum Electronics* **28**, 1–23 (2022).
